# When Cigarette Sales Suddenly Become Illegal: Evidence From an Online Survey of South African Smokers During COVID-19 Lockdown

**DOI:** 10.1093/ntr/ntac067

**Published:** 2022-05-02

**Authors:** Kirsten van der Zee, Samantha Filby, Corné van Walbeek

**Affiliations:** Research Unit on the Economics of Excisable Products (REEP), School of Economics, University of Cape Town, Cape Town, South Africa

## Abstract

**Introduction:**

Despite high levels of illicit trade in the tobacco market, the South African government banned the sale of tobacco products in March 2020 as part of its COVID-19 response. The ban lasted five months. We assess how the ban affected the cigarette market for a sample of smokers by comparing the price, consumption, and competitive landscapes before (March), during (May and June), and after (September) the ban.

**Methods:**

We conducted three online surveys of cigarette smokers, asking about smoking behavior before, during, and after the ban. We use descriptive statistics and OLS regressions to estimate the impact of the ban on the South African cigarette market, focusing on the price of cigarettes.

**Results:**

Most smokers continued smoking despite the sales ban. During the ban, prices increased by over 240%. Purchases shifted away from the normally dominant brands of the multinational tobacco companies to local/regional producers. The covariates of price changed substantially during the sales ban, the most pronounced being inter-provincial effects. After the ban, the market shifted back to its preban state, with an overall increase in price of 3.6%.

**Conclusions:**

Cigarette sales continued despite the sales ban, further entrenching an already large illicit market. Had the government substantially increased the excise tax, rather than banned the sale of tobacco products, it would have achieved a similar public health outcome, received more revenue, and presumably not further entrenched the illicit market.

**Implications:**

South Africa temporarily banned the sale of tobacco as part of its COVID-19 response. Despite the ban, the sale of cigarettes did not cease; rather, it caused major disruption to the cigarette market. The ban inadvertently benefited manufacturers who were previously disproportionately involved in illicit activities; these manufacturers increased their market share even after the ban was lifted. The ban may have further entrenched South Africa’s already large illicit market. Our results show that there are unintended consequences associated with a temporary ban on the sale of cigarettes.

## Introduction

Between 27 March 2020 and 17 August 2020, the sale of tobacco products was banned in South Africa as part of the country’s COVID-19 response. The prohibition was implemented on the presumption that smokers were more likely to develop severe illnesses and thus place extra pressure on the health system.^1^ Even though the sale of tobacco products was officially banned in South Africa, many smokers were able to access cigarettes during the sales ban, albeit at highly inflated prices.^2^ In this paper, we analyze the covariates of the price of cigarettes before, during, and after the sales ban to understand how the South African cigarette market changed in response to this policy decision.

Historically, the cigarette market in South Africa was dominated by multinational corporations, with British American Tobacco (BAT) having more than 90% of the market share in the early 21^st^ century.^3^ However, after 2010 smaller regional cigarette producers have entered the market, gradually reducing the market share of the multinationals.^4^ The new entrants undercut the multinationals’ retail prices and sold a large proportion of their products through informal outlets, such as street vendors and spaza shops. A consistent claim by the multinationals was that the new entrants evaded the excise tax, giving them an unfair advantage in the market.^5^ These claims have been denied by the new entrants, but surveys, both by the multinationals and by independent researchers, suggest that a large proportion of the new entrants’ products are sold at prices that are so low that the full tax amount could not have been paid.^2,5–8^

Independent studies show that South Africa’s illicit market increased sharply after 2010.^8^ The illicit share of the total cigarette market breached 10% for the first time in 2010. In 2015 the newly-appointed Commissioner of the South African Revenue Service (SARS), Tom Moyane, acting on mischievous information, abolished the special units that investigated the illicit cigarette market. Tobacco companies, which previously were closely monitored by SARS, became unsupervised. In the aftermath of this damage to SARS’s enforcement capacity, the illicit trade in cigarettes rose to approximately 30% of the total market in 2017.^6,8,9^

In August 2018, the incoming acting SARS Commissioner, Mark Kingon, announced the creation of the Illicit Economy Unit, aimed at addressing, among other things, the illicit trade in cigarettes.^10^ Despite this positive step and modest successes in reducing the illicit cigarette market in 2019,^11^ the illicit market was still firmly entrenched when the sales ban was announced in March 2020.

In response to the tobacco sales ban, we conducted three online surveys of smokers. Two of these surveys were conducted while the ban was in place and the third was conducted after the ban was lifted. Amongst other things, we wanted to understand how the cigarette market operated during the lockdown, and how prices behaved, using standard econometric techniques.

## Methods

The three surveys ran between 29 April and 11 May (round 1, *N* = 12 204), 4 and 19 June (round 2, *N* = 23 631), and 16 September and 5 October 2020 (round 3, *N* = 3766). The first two rounds were cross-sectional, whereas the third round was a longitudinal sub-set of round two respondents (those who agreed to be contacted again, and provided contact details). To be eligible for any of the three rounds, respondents had to be at least 18 years old and had to have smoked cigarettes in the week before the sales ban started. The surveys were conducted in English. Ethics clearance was granted by the Faculty of Commerce’s Ethics in Research Committee (numbers REC 2020/04/024, REC 2020/06/002, and REC 2020/09/003).

We used two platforms to host the questionnaire: the SurveyMonkey website and the Moya Messenger mobile application. Only one response was allowed per device. Moya is a data-/airtime-free instant messaging service, which allowed us to target lower-income respondents. The surveys were publicized through paid advertising on Twitter, and via an email sent to all people who signed a petition calling for the ban to be removed on the website change.org.

The questionnaire was broadly the same for the first two rounds, with sections asking about quitting behavior (in response to the ban), preban smoking behavior (consumption per day, purchasing behavior, brands purchased), information on whether they stocked up on cigarettes before the ban, consumption, and purchasing during lockdown, perceptions of the sales ban, and demographics. The third (substantially shorter) survey focused on respondents’ postban purchasing behavior, and whether they had purchased alternative tobacco products during the ban. The questionnaires and data can be found on DataFirst.^12^

Since we used an online survey tool, the data collection process was not designed to be representative of the national smoking population. We substantially over-sampled female smokers and white smokers. The average preban cigarette consumption in our sample is 15.5 cigarettes per day, whereas, nationally, cigarette consumption among smokers is eight sticks per day,^9^ indicating that we also oversampled heavier smokers.

We first provide descriptive statistics on the change in the retail price during and after the sales ban, followed by an econometric analysis. For the price analysis, we regressed the logarithm of the price per stick, against a number of covariates, for each of the three periods (i.e. before, during, and after the ban). Covariates include (1) the logarithm of cigarette consumption, (2) whether the respondent purchased a multinational or nonmultinational cigarette brand, (3) type of retail outlet where smokers purchased cigarettes, (4) packaging type (single sticks, 20-pack, carton, or other), (5) demographic and socio-economic variables (race, gender, and educational level), (6) duration of smoking, (7) geographic variables (province and area type), and (8) household income bracket.

Because most of these covariates are dummy variables, relatively small coefficients can be interpreted as percentage differences relative to the base scenario. However, for large coefficients, the effect of the variable becomes distorted and we calculate the percentage difference (relative to the base scenario) using the following formula:


$$\begin{array}{l} Percentage\;dif\;ference=exp\left(\beta \right)1 \end{array}$$ (1)

where β is the estimated regression coefficient. Both the estimated coefficients and the percentage differences are reported.

Since the dependent variable in the regressions is price and consumption is an independent variable, consumption may be endogenous, because high prices reduce consumption, and vice versa. For this reason, we test the regressions for endogeneity, using the Hausman endogeneity test. We use “smoking duration in years” as an instrumental variable for consumption, on the basis that it is reasonable to believe that the longer a smoker smokes, the more addicted they will become, thus the higher their consumption will be. In all three periods, we reject the null hypothesis that the instrument is weak, giving us confidence in our instrument. Where we find evidence of endogeneity from the Hausman test, we report the results from both OLS and the second stage of 2SLS.

For each round, price information was determined using the same two questions, the first asking the packaging type that the respondent had purchased (single stick, 10-pack, 20-pack, 30-pack or carton of 200), and the second asking how much they paid for each unit of the packaging type selected. This format is standard for tobacco questionnaires.^13^ Per-stick prices were obtained by dividing the unit price by the number of sticks per reported packaging type.

Several outliers were found in the price data, indicating that some respondents had incorrectly interpreted the price questions. In the appendix we describe the rules and principles that we followed in order to correct obvious reporting errors.

## Results

### Descriptive Statistics

About 9% of prelockdown smokers in the sample reported successfully quitting smoking during the tobacco sales ban. For continuing smokers, daily consumption decreased by an average of 18% between March and June. About 93% of respondents who continued smoking during lockdown indicated that they had purchased cigarettes during the lockdown. The analysis below focuses on the market that served these continuing smokers.

**Figure 1.**
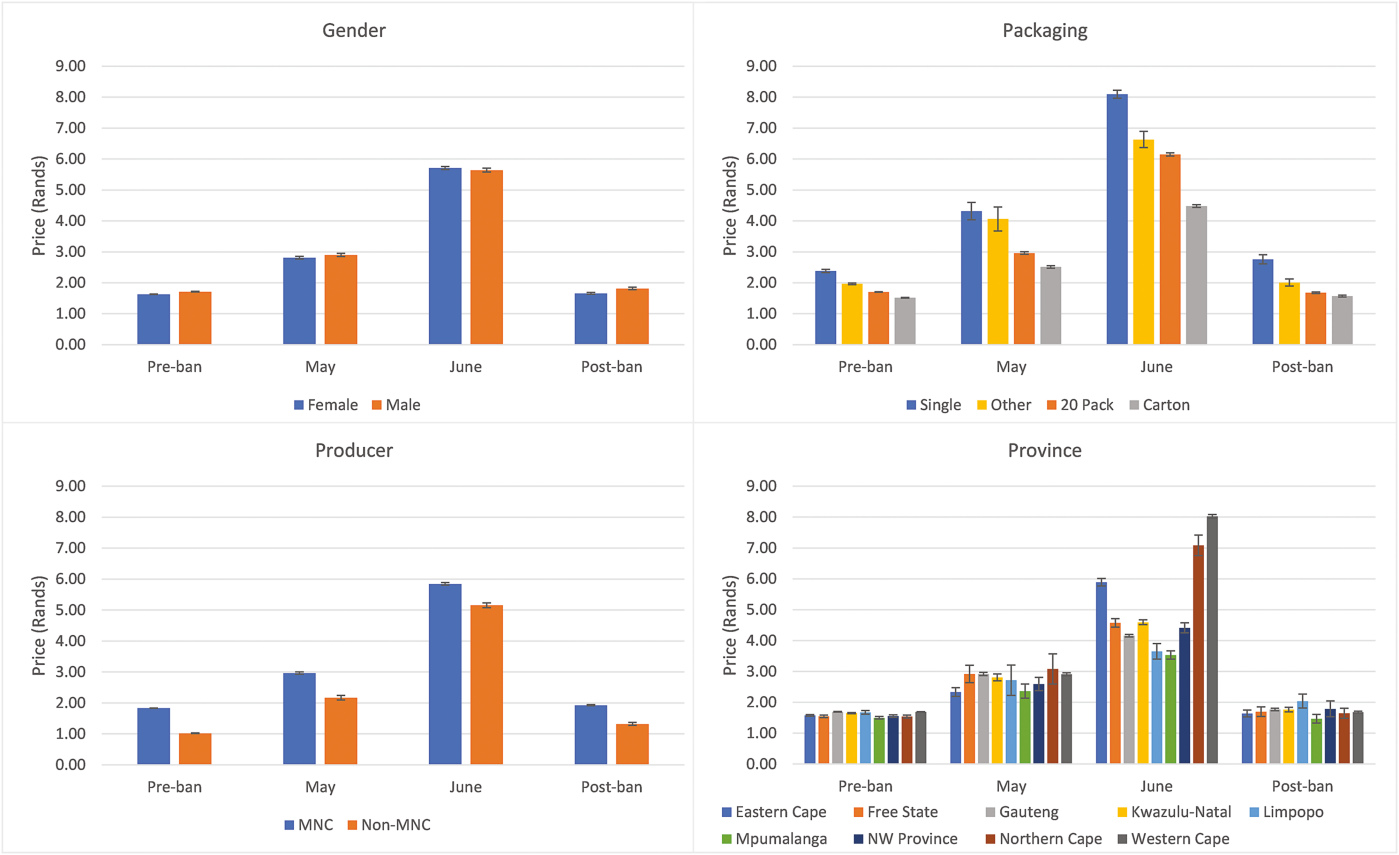
Mean price per stick by period and group. For packaging, “Other” includes 10- and 30-packs. MNC (multinational company) status is determined using the reported brand purchased. 95% confidence intervals displayed.

Figure 1 shows the average price per stick for various subgroups of the sample, before, during, and after the sales ban. Prior to lockdown, the average price in the sample was R1.67 per stick (R33 or 1.89 USD per 20-pack). By May the price had increased to R2.86 per stick (R57 or 3.26 USD per pack) and by June it had increased to R5.69 per stick (R114 or 6.51 USD per pack). Thus, the average retail price increased by more than 240% between March and June.

This price increase varied substantially by gender of the respondent, packaging type, manufacturer type (multinational (MNC) or nonmultinational (non-MNC)), and geographic region. Between March and June, the biggest price increases were experienced by females (250%) and for cigarettes sold in cartons (200 cigarettes) (260%). Non-MNC prices increased significantly more than MNC prices (410% compared to 219%). Non-MNC cigarettes were much cheaper at the outset, at an average of R1.02 per stick in March, compared to R1.83 per stick for MNC brands. Prices increased most in the Western Cape (374%), the Northern Cape (360%), and the Eastern Cape (273%).

Prices roughly returned to their preban levels after the ban was lifted. Overall, prices increased from R1.67 in March to R1.73 per stick in September, an average increase of 3.6%. The largest price increase was for non-MNC cigarettes, whose prices increased by 30%, from R1.02 per stick to R1.32 per stick. The average price of single sticks increased by 16%, from R2.38 to R2.76 per stick.

### Market Shares

**Figure 2.**
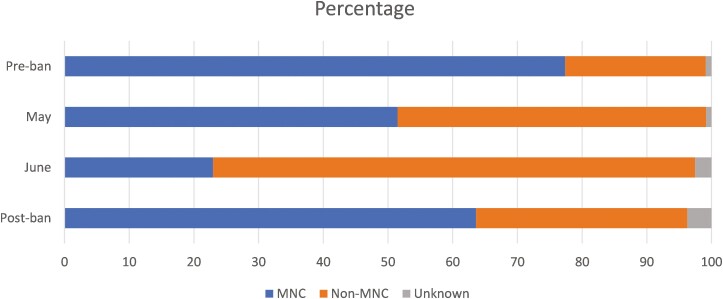
Sample market share, pre, during and postban. MNC (multinational company) status is determined using the reported brand purchased.

To illustrate how the brand landscape evolved over the pre-, during- and postban periods, Figure 2 presents the sample market shares for MNC and non-MNC brands for each period, weighted by cigarette consumption. It shows that, before the ban, MNC brands were by far the most popular choice, accounting for 77% of all cigarettes purchased by respondents, compared to the 22% for non-MNC brands.

During lockdown, however, market shares changed completely. By May, MNC brands made up 51% of the sample market, and this share dropped to less than 23% by June. By June, non-MNC brands were dominating the market, accounting for almost 75% of cigarettes bought by respondents in the sample.

In the postlockdown period, MNCs recovered some of their prelockdown market share, achieving nearly 64% of the sample market. However, this equates to an overall loss of 18% (or 14 percentage points) of their preban market share. By September, non-MNCs had gained 50% (or 11 percentage points) on their prelockdown (March) market share.

### Regression Analysis

From the descriptive statistics, we observe a large increase in prices during the ban, followed by a large decrease in prices after the ban. In this section we use regression analysis to estimate the impact of different covariates on the price of cigarettes (Supplementary Table 1), and how these impacts differed in each of the three periods. For the preban period we report on the 2SLS results, while for the other two periods we report on the OLS results since we did not detect endogeneity in these regressions.

For the preban period, respondents who smoked more cigarettes per day typically paid a slightly lower price for their cigarettes. In fact, a 1% higher consumption per day was associated with a 0.14% lower price. MNC brands were on average 73% more expensive than non-MNC brands. The prices of cigarettes bought at formal outlets and wholesalers were 10.6% and 5.4% higher, respectively, than cigarettes sold at informal convenience stores. Prices also varied significantly by packaging type. Single sticks were 45.1% more expensive, on average, than 20-packs. “Other” packaging (i.e., 10- and 30-packs) were 15.5% more expensive, while cartons were 3.9% less expensive than 20-packs.

There was some variation in the preban price across provinces, but these differences were small. Cigarettes bought in informal areas (townships and informal settlements) were 2.3% more expensive than those bought in urban areas. Smokers with higher incomes bought more expensive cigarettes.

Compared to the preban period, there were some major differences in the coefficients on the covariates of price during the ban. During the ban, smokers who smoked more cigarettes per day paid *more* for their cigarettes; a 1% increase in consumption was associated with an increase of 0.04% in price. MNC brands were still more expensive than non-MNC, but the magnitude of the price difference became smaller, with MNC prices being only 17% more expensive than non-MNC brands.

The outlet landscape changed significantly as traditional (formal) retail outlets were unable to sell cigarettes. Store types that were previously less used became more important. Compared to informal convenience stores (the base category), street vendors charged the highest prices (6.9% higher), followed by other categories (5.3% higher) and online platforms (4.8% higher). Cigarettes bought from family/friends were 1% cheaper than from informal convenience stores. Single sticks were the most expensive and cartons the cheapest, but the relative price differences changed. During the ban, cartons were, on average, 14.5% cheaper than 20-packs (compared to 3.9% cheaper preban) and singles were 31.4% more expensive, on average, than 20-packs (compared to 45.1% preban).

While some interprovincial variation existed preban, these differences became much greater during the ban. Relative to Gauteng (the base category), the highest prices were recorded in the Western Cape (83.7% higher), the Northern Cape (53.4% higher), and the Eastern Cape (36.9% higher). The lowest prices were in Limpopo (19.8% lower than Gauteng) and Mpumalanga (15.2% lower). While prices were higher on average in informal areas than in urban areas prior to the ban, they became 2.5% lower in informal areas during the ban.

Smokers in higher income categories still paid more for cigarettes during the ban, but the magnitude of the difference became smaller (the richest smokers paid 8.3% more for cigarettes than the poorest, compared to 13.8% in the preban period).

Lastly, the negative coefficient on age implies that older smokers paid less for cigarettes than their younger counterparts during the ban.

In the postban period, the coefficients on the covariates of price roughly reverted to their preban coefficients, with some key differences. While price and consumption are again negatively correlated, this correlation is not significant, as it had been preban. The difference in price between MNC and non-MNC brands is smaller than in the preban period (53.3% compared to 73.0% preban), indicating that the range of prices across manufacturing category has reduced. Price differences by outlet type are again similar to what they were before the ban, generally with slightly smaller magnitudes. For example, formal outlets still sell at prices significantly higher than informal convenience stores, but the difference is now only 6.8%, whereas it was 10.6% in the preban period.

For packaging, the difference in price between single sticks and 20-packs became larger in the postban period (61.1% higher on average, compared to 45.1% preban). Cartons are on average 6.6% cheaper per cigarette than 20-packs.

Whereas regional differences in price were very pronounced during the ban, we observed no significant differences in price across the provinces postban. Prices in informal areas are substantially higher than in urban areas, and this difference has grown, relative to the preban period (8.2% higher, relative to 2.3% preban).

The difference in price by income group is very similar in the postban period to what it was in the preban period, with the price paid by the richest smokers being on average 12.9% more (compared to 14.8% in the preban period) than the price paid by the poorest smokers.

## Discussion

The cigarette sales ban was largely unsuccessful in preventing smokers from purchasing cigarettes on the illicit market. From the outset, smokers knew that the sales ban was temporary. Less than 10% of smokers in our sample quit, despite the fact that the price of cigarettes more than tripled at the height of the ban. The fact that smokers persisted in consuming cigarettes despite these high prices can be explained by Becker and Murphy’s rational addiction hypothesis.^14^ According to this theory, an addict’s consumption response to a *temporary* change in price will be relatively small. Becker and Murphy argue that addicts will change their consumption more markedly if they expect that a price change will be permanent. This was not the case for South Africa’s temporary sales ban. For this reason, most smokers decided to bear these short-term exorbitant prices, rather than quit smoking. The fact that Covid-19 created numerous anxieties and stressors may have discouraged many smokers from quitting during this time and may even have caused some people to smoke more.

Our results indicate substantial differences between the preban cigarette market and the illicit market that operated during the sales ban. Most smokers were forced to switch brands because their regular brand was not available. The price of cigarettes grew rapidly during the first two months of the sales ban, and reached unprecedented highs around the middle of June, with average prices being about 240% higher than before the ban.

Whereas the South African cigarette market was fairly homogeneous before the ban, with similar average cigarette prices across the nine provinces, the market became increasingly fragmented during the sales ban. At the height of the ban, there were substantial differences in cigarette prices between the provinces. Provinces closer to Gauteng, the economic heartland of the country and home to many of the cigarette manufacturing plants, experienced substantially lower price increases than the more remote provinces. These regional differences in prices were probably caused by the restrictions on inter- and intra-provincial travel during the ban.

During the sales ban, the competitive structure changed substantially. The multinationals had been losing market share to the nonmultinationals even before the sales ban.^15^ The sales ban greatly accelerated this process. People who had traditionally smoked multinational brands were suddenly forced to buy whatever brand was available, and these were typically produced by the nonmultinationals.

Both local and multinational tobacco companies have been accused of being involved in a variety of illegal activities in South Africa, but when it comes to selling very cheap (and thus likely illicit) cigarettes, the nonmultinationals have a substantially larger presence in the market.^16^ Unless the nonmultinationals become more tax-compliant, either of their own volition or because they are monitored more closely by SARS, history suggests that the prevalence of cheap, untaxed cigarettes will increase when the market share of nonmultinationals increases. This may have negative implications for public health.

### Study Limitations

As mentioned in the methods section, we did not have a particular sampling strategy, and the resultant sample over-represented white smokers, females, and heavier smokers. We also promoted our survey on a petition site, which called for the end of the ban, which may have resulted in the data being skewed towards smokers who felt particularly strongly about the ban. Because the survey was done online, poorer and less computer-literate South Africans are under-represented because they have less access to the internet than more affluent groups.

Based on discussions with experts in sampling design, we were persuaded that weighting the data will not solve the representativity problem. For example, even though we can weight our sample of Africans up to national proportions, this will not give a representative picture of the full spectrum of African smokers, because very poor Africans were not sufficiently captured in our sample. Therefore, we decided not to weight the data, but to report the results from the sample we surveyed. We, therefore, do not attempt to claim national representativity.

We can, however, draw on recent findings from a broadly nationally representative survey, the National Income Dynamics Study—Coronavirus Rapid Mobile Survey (NIDS-CRAM). NIDS-CRAM is a five-wave panel survey that assesses the impact of COVID-19 on various socio-economic outcomes. The third wave of NIDS-CRAM included a cigarette module. Findings from this data suggest that approximately 7.9% of smokers quit during the ban period (compared to the 9% found in this paper), and that real cigarette prices increased to R110 per 20-pack at their highest point during the sales ban (compared to R114 in this paper).^17^ The fact that these and other estimates from NIDS-CRAM are similar to our findings gives us confidence that, while we cannot claim to be nationally representative, the REEP surveys give us useful and important insights into the responses of smokers and the market to this unprecedented sales ban.

## Supplementary Material

A Contributorship Form detailing each author’s specific involvement with this content, as well as any supplementary data, are available online at https://academic.oup.com/ntr.

ntac067_suppl_Supplementary_Appendix_A

ntac067_suppl_Supplementary_Table

ntac067_suppl_Supplementary_Taxonomy_Form

## Data Availability

Data are available at https://www.datafirst.uct.ac.za/dataportal/index.php/catalog/878
