# Supplementary material for: When Cigarette Sales Suddenly Become Illegal: Evidence From an Online Survey of South African Smokers During COVID-19 Lockdown
Source: Nicotine Tob Res. 2022 May 2;25(2):325–30. doi: 10.1093/ntr/ntac067 (PMC9383820; doi:10.1093/ntr/ntac067)
Supplement: ntac067_suppl_Supplementary_Appendix_A [file ntac067_suppl_supplementary_appendix_a.docx]

**APPENDIX A**

Several outliers were found in the price data, indicating that some respondents had incorrectly interpreted the price questions. A common error occurred when respondents reported the incorrect packaging type for the reported price, for example a price of R300 (about 17.14 USD)^[[1]](#footnote-1)^ for a pack of 20 cigarettes in the pre-lockdown period. Data on cigarette prices before the ban strongly suggest that this user misinterpreted the 20-pack option as a carton of 200 cigarettes. In cases where obvious errors such as these were made, we followed clearly defined correction rules, for example, reallocating this “20-pack” to be recorded as a carton of 200, and using this to calculate the per stick price. Because there is substantial variation in prices between brands, brands were also considered when cleaning the prices.

For the pre-lockdown period, we flagged any of the following prices: single stick prices of less than R0.50 (USD 0.03) or more than R4.50 (USD 0.26), 10-pack prices of less than R5 (USD 0.29) or more than R30 (USD 1.71), 20-pack prices of less than R8 (USD 0.46) or more than R60 (USD 3.43), 30-pack prices of less than R12 (USD 0.69) or more than R90 (USD 5.14), and carton prices of less than R50 (USD 2.86) or more than R400 (USD 22.86). These price points are based on South African cigarette pricing data [18].

Because the tobacco ban was unprecedented, we could not look to existing data to inform price rules during the sales ban. Anecdotal evidence from desperate smokers indicated that they were willing to pay exorbitant prices for cigarettes. Based on a thorough analysis of the raw data and by assessing media reports on cigarette prices [19], we applied the assumption that reported prices as high as R25 (USD 1.43) per single stick, R150 (USD 8.57) for a 10-pack, R300 (USD 17.14) for a 20-pack, R450 (USD 25.71) for a 30-pack, and R3000 (USD 171.43) for a carton of 200 cigarettes were plausible. Prices outside of these are flagged and checked for errors, or omitted.

Again, one could not use previous data to inform price rules for the post-ban period, since there was no precedent for how the tobacco market would settle once the ban was lifted. Our price cleaning was based on a thorough analysis of the raw data. We flagged the following prices: single stick prices of more than R6 (USD 0.34), 10-pack prices of less than R5 (USD 0.29) or more than R50 (USD 2.86), 20-pack prices of less than R12 (USD 0.69) or more than R65 (USD 3.71), and carton prices of less than R60 (USD 3.43) or more than R600 (USD 34.29). There were no 30-pack prices that were flagged as this sample was very small (N = 25).

1. The rand/dollar exchange rate was very volatile during 2020, ranging from R14.03 to R19.26 to the USD. We use a value of R17.50, since this was the average exchange rate during the sales ban period. [↑](#footnote-ref-1)
