# Supplementary material for: When Cigarette Sales Suddenly Become Illegal: Evidence From an Online Survey of South African Smokers During COVID-19 Lockdown
Source: Nicotine Tob Res. 2022 May 2;25(2):325–30. doi: 10.1093/ntr/ntac067 (PMC9383820; doi:10.1093/ntr/ntac067)
Supplement: ntac067_suppl_Supplementary_Table [file ntac067_suppl_supplementary_table.docx]

Supplementary Table 1: Covariates of price, pre-, during and post-ban

|  | **Pre-ban** | | | **During ban** | | **Post-ban** | |
| --- | --- | --- | --- | --- | --- | --- | --- |
|  | **Log(stick)** | **Log(stick)** | **% difference** | **Log(stick)** | **% difference** | **Log(stick)** | **% difference** |
|  | **OLS** | **IV** | **IV** | **OLS** | **OLS** | **OLS** | **OLS** |
| **Log(pre-LD Consumption) / log(LD consumption)** | -0.019*** | -0.137*** |  | 0.037*** |  | -0.013 |  |
|  | -5.27 | -4.82 |  | 7.61 |  | -1.33 |  |
| **Producer (Base: non-MNC)** |  |  |  |  |  |  |  |
| MNC | 0.553*** | 0.548*** | 0.730 | 0.158*** | 0.171 | 0.427*** | 0.533 |
|  | 80.72 | 77.79 |  | 18.98 |  | 29.56 |  |
| **Outlet type (Base: Informal convenience)** |  |  |  |  |  |  |  |
| Formal | 0.105*** | 0.101*** | 0.106 | -0.018 | -0.018 | 0.066*** | 0.068 |
|  | 18.08 | 16.52 |  | -0.55 |  | 4.27 |  |
| Street | -0.012 | -0.015 | -0.015 | 0.067*** | 0.069 | 0.084* | 0.088 |
|  | -0.45 | -0.55 |  | 7.69 |  | 1.78 |  |
| Online | -0.112 | -0.179 | -0.164 | 0.047*** | 0.048 | 0.015 | 0.015 |
|  | -0.37 | -0.56 |  | 5.18 |  | 0.23 |  |
| Friends/Family | 0.123* | 0.077 | 0.080 | -0.01 | -0.010 | 0.274*** | 0.315 |
|  | 1.76 | 1.09 |  | -1.34 |  | 3.02 |  |
| Acquaintances (essential workers) |  |  | 0.000 | 0.025 | 0.025 | 0.118 | 0.125 |
|  |  |  |  | 1.27 |  | 1.01 |  |
| Wholesaler | 0.055*** | 0.053*** | 0.054 | 0.001 | 0.001 | 0.037 | 0.038 |
|  | 4.49 | 4.25 |  | 0.02 |  | 1.36 |  |
| Other | -0.003 | -0.005 | -0.005 | 0.052*** | 0.053 | -0.019 | -0.010 |
|  | -0.17 | -0.32 |  | 4.08 |  | -0.21 |  |
| **Packaging (Base: 20 pack)** |  |  |  |  |  |  |  |
| Single | 0.419*** | 0.372*** | 0.451 | 0.273*** | 0.314 | 0.477*** | 0.611 |
|  | 26.71 | 18.67 |  | 24.13 |  | 12.61 |  |
| Carton | -0.066*** | -0.040*** | -0.039 | -0.157*** | -0.145 | -0.068*** | -0.066 |
|  | -16.09 | -5.33 |  | -21.03 |  | -5.82 |  |
| Other | 0.152*** | 0.144*** | 0.155 | 0.162*** | 0.176 | 0.112*** | 0.119 |
|  | 13.49 | 12.18 |  | 4.63 |  | 2.66 |  |
| **Male** | -0.005 | 0.006 | 0.006 | -0.023*** | -0.023 | 0.01 | 0.010 |
|  | -1.36 | 1.31 |  | -3.98 |  | 0.87 |  |
| **Province (Base: Gauteng)** |  |  |  |  |  |  |  |
| Eastern Cape | -0.012 | -0.018** | -0.018 | 0.314*** | 0.369 | -0.033 | -0.032 |
|  | -1.53 | -2.21 |  | 23.79 |  | -1.36 |  |
| Free State | -0.033*** | -0.029** | -0.029 | 0.059*** | 0.061 | -0.031 | -0.031 |
|  | -2.82 | -2.5 |  | 3.81 |  | -0.91 |  |
| KwaZulu-Natal | -0.013** | -0.016*** | -0.016 | 0.044*** | 0.045 | -0.01 | -0.010 |
|  | -2.19 | -2.64 |  | 4.78 |  | -0.55 |  |
| Limpopo | 0.023 | 0.026 | 0.026 | -0.221*** | -0.198 | 0.037 | 0.038 |
|  | 1.33 | 1.45 |  | -7.18 |  | 0.87 |  |
| Mpumalanga | 0.004 | 0.004 | 0.004 | -0.165*** | -0.152 | -0.06 | -0.058 |
|  | 0.3 | 0.33 |  | -8.99 |  | -1.34 |  |
| North-West Province | -0.015 | -0.01 | -0.010 | 0.076*** | 0.079 | -0.023 | -0.023 |
|  | -1.15 | -0.79 |  | 4.24 |  | -0.48 |  |
| Northern Cape | -0.052*** | -0.050*** | -0.049 | 0.428*** | 0.534 | 0.006 | 0.006 |
|  | -3.56 | -3.31 |  | 17.76 |  | 0.13 |  |
| Western Cape | 0.011** | 0.008* | 0.008 | 0.608*** | 0.837 | -0.017 | -0.017 |
|  | 2.54 | 1.75 |  | 77.05 |  | -1.35 |  |
| **Area type (Base: urban)** |  |  |  |  |  |  |  |
| Rural | -0.004 | -0.001 | -0.001 | -0.013 | -0.013 | 0.02 | 0.020 |
|  | -0.55 | -0.09 |  | -1.24 |  | 1.08 |  |
| Informal | 0.024** | 0.023** | 0.023 | -0.025* | -0.025 | 0.079*** | 0.082 |
|  | 2.5 | 2.29 |  | -1.67 |  | 2.89 |  |
| **Income category (Base: R0 – R6 400)** |  |  |  |  |  |  |  |
| R6 401 – R51 200 | 0.040*** | 0.053*** | 0.054 | 0.019** | 0.019 | 0.048*** | 0.049 |
|  | 6.58 | 7.65 |  | 2.26 |  | 2.81 |  |
| R51 201 and more | 0.126*** | 0.138*** | 0.148 | 0.080*** | 0.083 | 0.121*** | 0.129 |
|  | 17.23 | 17.25 |  | 6.73 |  | 5.61 |  |
| **Age** | 0.001*** | 0.002*** |  | -0.003*** |  | 0.001 |  |
|  | 5.71 | 6.2 |  | -10.57 |  | 1.53 |  |
| **Constant** | 0.123 | 0.331*** | 0.392 | 1.518*** | 3.563 | 0.035 | 0.036 |
|  | 1.22 | 3.01 |  | 19.97 |  | 0.94 |  |
|  |  |  |  |  |  |  |  |
| **Observations** | 20,801 | 20,745 |  | 12,804 |  | 2,538 |  |
| **R-squared** | 0.573 | 0.549 |  | 0.577 |  | 0.519 |  |

Notes: The dependent variable is the natural logarithm of the price per stick (pre-, during, and post-ban). Log(cigarette consumption) is the natural log of the average per day consumption, in the corresponding period. T-statistics are displayed below the coefficients. Significance stars represent: *** p<0.01, ** p<0.05, * p<0.1. Each regression was tested for endogeneity (Hausman test) in consumption, using smoking duration (in years) as an IV. The test reveals endogeneity for the pre-ban regression (p=0.000) but not for the during (p=0.4229) or post-ban regressions (p=0.1697). For this reason, OLS and 2SLS estimates are reported for the pre-ban regression. In all three regressions we reject the null hypothesis for weak instruments, at the 95% level.
Ethnic group and educational level are included as control variables but are not reported due to space constraints. MNC stands for multinational company. For packaging, “Other” includes 10- and 30-packs. For outlet type, “Formal” includes formal retailers, petrol stations, liquor stores, and tobacco shops; “Informal convenience” includes spaza shops, cafes and house shops; “Street” includes street vendors and car guards, and “Online” includes WhatsApp and other online platforms. For area type, “Urban” includes city, suburb and town; “Rural” includes farm and rural, and “Informal” includes informal settlements and townships.
